# Psychometric Properties of the Cardiac Rehabilitation Self‐Efficacy Questionnaire in Heart Failure Patients: A Cross Sectional Study Using Health Action Process Approach Perspective

**DOI:** 10.1002/hsr2.70188

**Published:** 2024-11-06

**Authors:** Zahra Fallah, Awat Feizi, Masoumeh Sadeghi, Mohammad Mahdi Hadavi, Hossein Shahnazi

**Affiliations:** ^1^ Department of Health Education and Promotion School of Health, Isfahan University of Medical Sciences Isfahan Iran; ^2^ Department of Biostatistics and Epidemiology School of Health, Isfahan University of Medical Sciences Isfahan Iran; ^3^ Cardiac Rehabilitation Research Center, Isfahan University of Medical Sciences Isfahan Iran

**Keywords:** exercise, health action process approach, psychometric, rehabilitation, self‐efficacy

## Abstract

**Background and Aim:**

The present study aimed to develop and psychometrically evaluate the exercise self‐efficacy tool within the Health Action Process Approach (HAPA) framework among heart failure patients undergoing cardiac rehabilitation.

**Methods:**

This study assessed 205 patients who had suffered heart attacks at the Shahid Chamran Cardiac Rehabilitation Center of Isfahan. Exploratory factor analysis (EFA) was employed to evaluate the construct. Internal reliability was determined using Cronbach's alpha. Additionally, external reliability was measured through a test‐retest approach.

**Results:**

EFA identified four factors within the self‐efficacy questions (task self‐efficacy 1, task self‐efficacy 2, coping self‐efficacy, and recovery self‐efficacy), which accounted for 70.1% of the total variance explained. The Cronbach's alpha coefficients were as follows: 0.836 for the first factor, 0.896 for the second factor, 0.921 for the third factor, 0.914 for the fourth factor, and 0.9 for the overall instrument. The intra‐class correlation coefficient was 0.901 for the first factor, 0.887 for the second factor, 0.826 for the third factor, and 0.885 for the fourth factor.

**Conclusion:**

The cardiac rehabilitation self‐efficacy questionnaire exhibited high validity, reliability, and desirable item commonalities. Therefore, it can be effectively employed in pertinent HAPA‐based studies involving heart failure patients.

## 1 Introduction

Cardiac sports rehabilitation constitutes an effective strategy for enhancing the quality of life, augmenting functional capacity, and optimizing VO2 levels in heart failure patients. Despite the well‐documented advantages of exercise training in rehabilitation, there has been limited participation by cardiovascular patients [1, 2, 3, 4]. Reports indicate that only 30% of individuals in developed countries and 28% in Iran engage in such programs [5, 6, 7]. Given the global prevalence of cardiovascular diseases, which account for 31% of all deaths, with over three‐quarters occurring in low‐income countries, it is imperative to implement effective measures to increase patient engagement in rehabilitation programs [8, 9, 10].

Utilizing psychological processes is a practical approach for attracting patients to rehabilitation programs [11]. Self‐efficacy, a psychological construct, profoundly connects with various lifestyle‐related behaviors across various domains [12]. This construct delineates an individual's belief in their ability to perform a given behavior, closely intertwined with mental health [13].

According to Schwartz, distinct self‐efficacy beliefs are required at different stages of behavioral change, leading to the definition of specific types of self‐efficacy [13]. For instance, individuals may generally have confidence in their capacity to engage in physical activity but may lack confidence in restarting such activities following a setback [14]. The Health Action Process Approach (HAPA) provides a framework in which self‐efficacy is pivotal across different stages of adopting healthy lifestyle behaviors, including sports activities [15]. Self‐efficacy within the HAPA model involves a two‐step motivational and volitional process [16].

**Figure 1:**
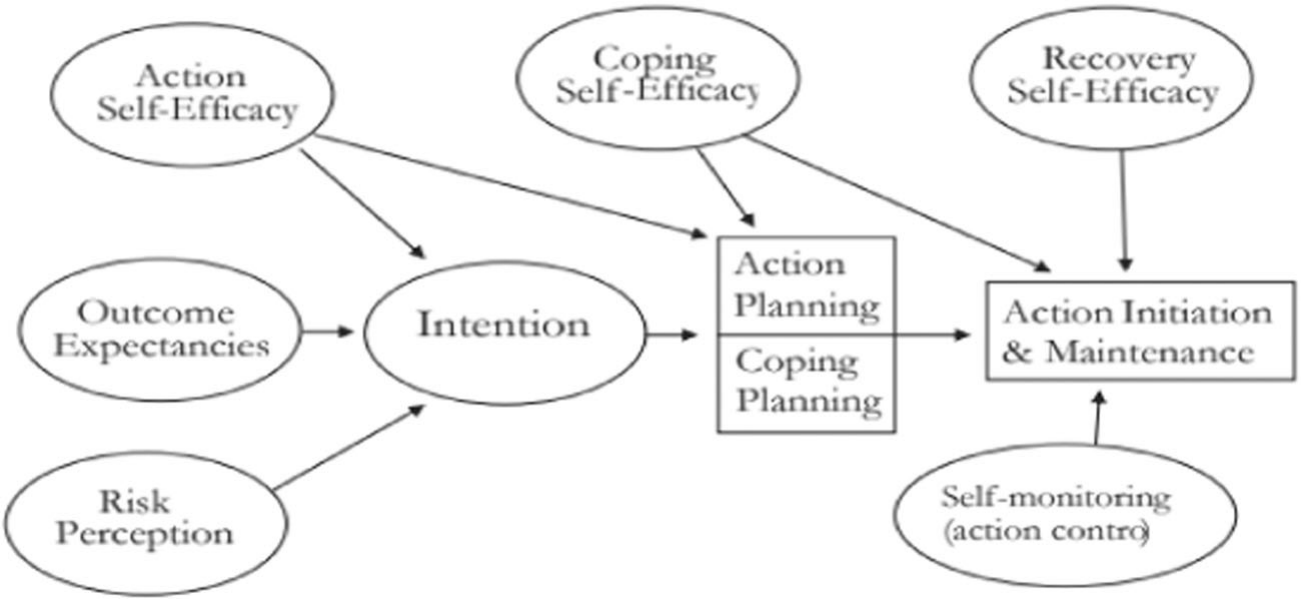
Health action process approach (Schwarzer, 1992, 2008).

Within the HAPA framework, three categories of self‐efficacy come into play at different stages [17]. Task self‐efficacy pertains to the stage where an individual is motivated to perform a task but has yet to take action. Coping self‐efficacy revolves around an individual's belief in coping with barriers that may arise after initiating the behavior and during the maintenance phase. Recovery self‐efficacy is the capacity to rebound and regain control following a failure. It reflects an individual's confidence in their ability to rectify the situation post‐failure and return to the correct behavior path [18, 19, 20] (Figure 1).

This distinction among various self‐efficacy beliefs has been substantiated at each stage of behavioral change [21]. Bandura advocates that the operationalization of self‐efficacy concepts should be grounded in a comprehensive analysis and a deep understanding of factors specific to a given behavior and performance issue [20]. Consequently, domain‐specific instruments must be developed, considering all facets of the domain, to yield a valid measurement. Moreover, the self‐efficacy scale should assess the patient's ability to master the situations and challenges inherent to a particular field of performance [17, 22].

The “health action process approach” (HAPA) framework helps us understand how individuals can succeed in different stages of behavior change, such as starting and continuing physical activity. In this framework, “self‐efficacy” is recognized as a key factor. Self‐efficacy refers to an individual's belief in their ability to perform a specific behavior. In other words, if a person feels they can effectively engage in exercise and overcome barriers, they are more likely to continue with the activity. For example, a patient who has suffered a heart attack and believes in their ability to start an exercise program may be more motivated to participate in cardiac rehabilitation programs. The HAPA framework illustrates how self‐efficacy plays a role at various stages, including motivation to start an activity (task self‐efficacy), the ability to cope with challenges (coping self‐efficacy), and confidence in recovering after setbacks (recovery self‐efficacy). Understanding self‐efficacy in this way can help us design more effective programs to engage patients in cardiac rehabilitation [22, 23].

Numerous tools have been adapted and validated in different languages and countries to assess exercise self‐efficacy. For example, Bandura's 18‐item exercise self‐efficacy (ESE) scale has received validation in Indonesia [24], Korea [25], Australia [26], and Iran [27]. It underwent psychometric evaluation in diabetic patients in Iran [28]. Nevertheless, previous tools may prove inadequate due to potential disparities in barriers and challenges encountered by diverse populations. Self‐efficacy must be thoroughly measured to comprehensively capture the broader and clearer psychological determinants, such as family support, pain, fatigue, lack of motivation, and disinterest in commencing exercise. Furthermore, cultural and social contexts within Iranian society should be considered when evaluating psychological constructs across different stages of rehabilitation, from initiation and maintenance to recovery following relapse.

This study addresses the question of whether it is possible to design a tool that possesses the necessary validity and reliability in the population of patients with myocardial infarction and measures various dimensions of self‐efficacy at different stages of the HAPA. Consequently, this study aims to develop and psychometrically evaluate an exercise self‐efficacy tool based on the HAPA within the context of heart failure patients undergoing cardiac rehabilitation.

## 2 Methods

### 2.1 Study Design and Participants

This cross‐sectional study aimed to develop and evaluate the psychometric properties of a rehabilitation self‐efficacy tool in heart failure patients in Isfahan City from January to June 2022. The researcher visited the Shahid Chamran Rehabilitation Center to achieve this objective, where eligible patients were carefully selected. Subsequently, the researcher completed the questionnaires after thoroughly explaining the research's purpose and obtaining written consent from the participants.

The inclusion criteria for participants encompassed individuals diagnosed with heart failure by medical professionals, possessing literacy skills and lacking musculoskeletal problems hindering rehabilitation. On the contrary, participants who did not consent to the study or had mental and psychological problems were excluded. Questionnaires were administered through both interviews and self‐reporting methodologies. The Ethical Committee of Isfahan University of Medical Sciences approved the research design with an ethical code IR. MUI. RESEARCH. REC.1399.755.

### 2.2 Designing Questionnaire Items

The initial step in developing the questionnaire items involved a comprehensive review of global studies concerning the exercise self‐efficacy of heart failure patients undergoing rehabilitation. Drawing insights from this literature review [13, 29, 30], a collection of 26 initial items was formulated in Persian. These items were meticulously constructed in alignment with various facets of self‐efficacy as delineated within the HAPA framework.

### 2.3 Content Validity Assessment

To ascertain qualitative content validity, five experts were engaged, consisting of four health education specialists and one cardiologist. They were tasked with meticulously examining the items, providing intricate oral feedback concerning grammar, word selection, item placement, and appropriateness of scoring. Subsequently, their invaluable insights were duly incorporated into the questionnaire.

The questionnaires were distributed among 13 experts, comprising 10 health education specialists, 1 cardiologist, and 2 physiotherapists. Their quantitative evaluation of the questionnaire content involved categorizing each item into one of three options: “Essential,” “Useful but not essential,” and “Not Necessary.” Additionally, the content validity ratio (CVR) was calculated using the following formula:

CVR=[ne−(N/2)]/(N/2)

Items with CVRs exceeding 0.51 were retained following Lawshe's ratio [31]. Furthermore, the expert panel was solicited for assessments of relevance (1 = irrelevant, 2 = partially relevant, 3 = relevant, 4 = completely relevant), simplicity (1 = non‐simple, 2 = relatively simple, 3 = simple, 4 = completely simple), and clarity (1 = Unclear, 2 = relatively straightforward, 3 = clear, 4 = completely clear) for each item. Subsequently, the Content Validity Index (CVI) was computed using the following formula:

$$\text{CVR}\;=\frac{\text{Number of}\;\text{experts who gave scores of}\;3\;\text{and}\;4\;\text{to items}}{\text{Total number of}\;\text{experts}}$$

Items garnering CVIs exceeding 0.79 were deemed acceptable. Items flagged by experts as unclear or ambiguous were subjected to reformulation, with their suggested improvements duly implemented.

### 2.4 Face Validity Assessment

Face validity was evaluated qualitatively through consultation with five experts, whose qualifications had been previously documented, and five patients from the rehabilitation center. Alongside comprehensive explanations, verbal feedback was solicited from these individuals regarding the comprehensibility of each questionnaire item. Any necessary refinements were promptly incorporated based on their invaluable input.

### 2.5 Construct Validity

In this investigation, a sample size comprising 205 patients suffering from heart failure was meticulously chosen. This sample size determination was done based on the referenced recommendations for exploratory factor analysis. It is suggested that the minimum sample size for factor analysis is equal to the number of items multiplied by 5–10 [32, 33] or Bujang et al. (2012) recommend that the required number of participants for EFA is between 3 and 10 people per item, or a total of 100–200 participants [34]. Based on our study objective we developed a new questionnaire for evaluating the rehabilitation self‐efficacy among patients with heart failure, accordingly for determining its construct or dimensions we employed the exploratory factor analysis (EFA) method [35]. Additionally, principal component analysis (PCA) served as the chosen technique for factor extraction. The appropriate number of factors was determined based on a combination of the scree plot analysis and the criterion of an eigenvalue exceeding 1. To enhance interpretability, orthogonal varimax rotation was employed for the derived factors.

Furthermore, the Kaiser‐Meyer‐Olkin (KMO) test was employed to assess the adequacy of the sampling, with a threshold value exceeding 0.7 serving as the benchmark for acceptability. To evaluate the suitability of the data for factor analysis, Bartlett's test of sphericity was also conducted, considering a p‐value of less than 0.05 as indicative of statistical significance. Subsequently, names were assigned to these factors based on the items that exhibited substantial loadings within each factor. The score of each subscale (factor) was quantified by multiplying the total loading of the individual items within the factor by their corresponding scores. These computed scores were then assigned to each participant in the study.

### 2.6 Reliability Assessment

#### 2.6.1 Internal Reliability

Cronbach's alpha index was calculated for each dimension and the entire questionnaire in the pilot group (*n* = 20) to determine internal reliability. Values greater than 0.70 were considered satisfactory [31].

#### 2.6.2 External Reliability

The test‐retest method was employed to assess external reliability. In this regard, the questionnaire was administered once more to 20 patients at intervals ranging from 10 days to 2 weeks. The intra‐class correlation coefficient (ICC) was computed using a two‐way mixed method with a confidence interval of 0.95. This coefficient was determined for each dimension and the entire questionnaire within the pilot group (*n* = 20). Values below 0.5 were categorized as weak, those within the range of 0.5–0.75 were considered moderate, and values falling between 0.75 and 0.9 were regarded as very good [31].

### 2.7 Additional Measurements and Statistical Analysis

Quantitative variables were reported utilizing the mean and standard deviation, whereas qualitative variables were expressed as numerical values and percentages. Statistical analyses were performed using SPSS 26 software (IBM Corp. Released 2019. IBM SPSS Statistics for Windows, Version 26.0. Armonk, NY: IBM Corp).

## 3 Results

The participants in this study consisted of heart failure patients with an average age of 64.44 years. Table 1 provides an overview of the demographic characteristics of the participants.

**Table 1:** Demographic characteristics of the participants.

Characteristics		Mean (SD) or Number (percent) *n* = 205
Age		64.4 (4.46)
Gender	Male	158 (78.6)
	Female	43 (21.4)
Marital status	Single	9 (4.5)
	Married	184 (90.5)
	Widow	8 (5)
Educational level	Primary school	49 (24.4)
	Intermediate and diploma	83 (41.29)
	College	69 (34.32)
Current job	Retired	61 (30.8)
	Housekeeper	31 (15.4)
	Employed	28 (14.1)
	Unemployed	6 (3)
	Other	51 (30)

### 3.1 Content and Face Validity

Experts evaluated the questionnaire items' simplicity, relevance, clarity, and necessity. A CVI greater than 0.7 was considered acceptable, leading to the exclusion of two items during this evaluation stage. The CVR exceeded 0.8 for all items, resulting in no removal of items at this stage. No issues or ambiguities were identified in the questionnaire, and all items were considered important and relevant during the assessment of face validity. Consequently, no changes were made to the content of the symptoms in the questionnaire (Table 2).

**Table 2:** Item Content Validity Index (I‐CVI), and Content Validity Ratio (CVR) values of the rehabilitation self‐efficacy questionnaire.

Component	Item	I‐CVI	CVR
1. Task self‐efficacy 1	How confident are you in doing your exercise training without interruption? Specify your confidence level for each period in minutes.		
	I am sure I can exercise 15 min per day.	1	1
	I am sure I can exercise 20 min per day.	1	1
	I am sure I can exercise 30 min per day.	1	1
	I am sure I can exercise 40 min per day.	1	1
	I am sure I can exercise 50 min per day.	1	1
	I am sure I can exercise 60 min or more per day.		
2. Task self‐efficacy 2	How many minutes can you do your exercises per day? Specify your confidence level for each day per week.	1	1
	I am sure I can exercise twice per week.	1	1
	I am sure I can exercise three times per week.	1	1
	I am sure I can exercise four times per week.	1	1
	I am sure I can exercise five times per week.	1	1
	I am sure I can exercise six times per week.	1	1
3. Maintenance self‐efficacy	How confident are you in doing your exercise training in the next 6 months under the following conditions?		
	Even if it takes me a long time to get used to exercising, I can do my exercise.	0.89	1
	Even if my progress in doing sports is slow, I can continue my exercise.	0.97	0.85
	Even in difficult situations such as fatigue, discomfort, and worry, I am unwilling to leave my exercise.	0.97	0.85
	Even if I do not see the results of exercising very soon, I will continue my exercise.	0.82	1
	Even if others do not accompany me, I continue my exercise.	0.92	1
	Even if I do not have a suitable place to exercise, I continue my exercise.	0.97	0.85
	Even if I feel physically tired and painful, I still find a way to exercise.	0.94	1
4. Recovery self‐efficacy	I am sure that I can start my exercise again even if…		
	I have not been active for a while due to illness and body pain.	0.94	1
	I have not been active due to fatigue and exercise difficulty.	0.97	1
	I have not been active for a while due to not being accompanied by my family.	0.97	1
	I have not been active for a while due to forgetfulness.	0.97	1
	I have not been active because of travel, partying, or a holiday.	1	1
	I have not been active for a while because of boredom.	1	1

### 3.2 Construct Validity

Regarding Factor Validity, the KMO test yielded a value of 0.88, indicating that the sample size was sufficient for conducting an EFA. Additionally, Bartlett's test confirmed the factorability of the data (*p* < *0.05*). Using varimax rotation, EFA extracted four factors from the self‐efficacy questions, explaining 70.1% of the variance. The extracted factors were named based on the general concept of the items as follows:

1. Coping Self‐Efficacy (Factor 1) included 7 items with a variance of 21.507, reflecting individuals' confidence levels in their ability to maintain exercise routines under challenging conditions (e.g., fatigue or lack of support from others) over the next 6 months.
2. Task Self‐Efficacy 2 (Factor 2) comprised 6 items with a variance of 17.72, indicating individuals' confidence in their ability to engage in uninterrupted exercise for prolonged periods during the day (30 min or more) and on specific days of the week (five times or more) over the next 6 months.
3. Recovery Self‐Efficacy (Factor 3) consisted of 6 items with a variance of 17.662, reflecting individuals' confidence in their capacity to resume an exercise program after an interruption (relapse) within the next 6 months.
4. Task Self‐Efficacy 1 (Factor 4) included five items with a variance of 13.212, representing individuals' confidence in their ability to engage in uninterrupted exercise for shorter periods during the day (10 and 20 min) and on a specific number of days (1–3 days a week) over the next 6 months.

Table 3 displays the factor loadings of the four factors extracted from the EFA for the 24 items.

**Table 3:** Factor loadings of the rehabilitation self‐efficacy scale to assess construct validity.

	Rotated component matrixa			
	Factor			
**Items**	Coping self‐efficacy	Task self‐efficacy 2	Recovery self‐efficacy	Task Sel efficacy 1
I am sure I can exercise 15 min per day.	—	—	—	0.748
I am sure I can exercise 20 min per day.	—	—	—	0.689
I am sure I can exercise 30 min per day.	—	0.703	—	—
I am sure I can exercise 40 min per day.	—	0.904	—	—
I am sure I can exercise 50 min per day.	—	0.928	—	—
I am sure I can exercise 60 min or more per day.	—	0.873	—	—
I am sure I can exercise twice per week.	—	—	—	0.799
I am sure I can exercise three times per week.	—	—	—	0.745
I am sure I can exercise four times per week.	—	—	—	0.528
I am sure I can exercise five times per week.	—	0.580	—	—
I am sure I can exercise six times per week.	—	0.551	—	—
Even if it takes me a long time to get used to exercising, I can do my exercise.	0.746	—	—	—
Even if my progress in doing sports is slow, I can continue my exercise.	0.787	—	—	—
Even in difficult situations such as fatigue, discomfort, and worry, I am unwilling to leave my exercise.	0.778	—	—	—
Even if I do not see the results of exercising very soon, I will continue my exercise.	0.812	—	—	—
Even if others do not accompany me, I continue my exercise.	0.799	—	—	—
Even if I do not have a suitable place to exercise, I continue my exercise.	0.823	—	—	—
Even if I feel physically tired and painful, I still find a way to exercise.	0.694	—	—	—
I am sure that I can start my exercise again even if…	—	—	0.581	—
I have not been active for a while due to illness and body pain.	—	—	0.808	—
I have not been active due to fatigue and exercise difficulty.	—	—	0.862	—
I have not been active for a while due to not being accompanied by my family.	—	—	0.864	—
I have not been active for a while due to forgetfulness.	—	—	0.847	—
I have not been active because of travel, partying, or a holiday.	—	—	0.881	—

*Note:* Exploratory factor analysis with Varimax rotation; Factor loadings < 0.4 are not shown for simplicity. Variance explained resulted from factor analysis.

### 3.3 Reliability Analysis

Cronbach's alpha yielded the following results for the respective factors: 0.922 for the first factor, 0.836 for the second factor, 0.914 for the third factor, 0.896 for the fourth factor, and 0.926 for the entire tool. Thus, the internal consistency among the factors was remarkably high and substantiated. Moreover, the ICC revealed values of 0.901 for the first factor, 0.887 for the second factor, 0.826 for the third factor, and 0.885 for the fourth factor, indicating commendable repeatability (Table 4).

**Table 4:** Results of internal reliability and test‐retest of the questionnaire.

Pilot sample *N* = 20					ICC	Total sample *N* = 205		
		Corrected Item‐ total correlation	Cronbachs alpha if item delete	alpha		Corrected Item‐ total correlation	Cronbachs alpha if item delete	alpha
Factor	item			0.828	0.887			0.836
Task self efficacy 1	1	0.764	0.754			0.585	0.821	
	2	0.716	0.766			0.634	0.804	
	3	0.721	0.789			0.650	0.802	
	4	0.742	0.772			0.772	0.763	
	5	0.400	0.887			0.612	0.822	
Task self efficacy 2	1	0.467	0.869	0.862	0.826	0.675	0.884	0.896
	2	0.775	0.817			0.822	0.861	
	3	0.812	0.810			0.840	0.858	
	4	0.705	0.830			0.762	0.871	
	5	0.625	0.845			0.644	0.890	
	6	0.561	0.856			0.584	0.898	
Coping self‐efficacy	1	0.451	0.887	0.881	0.901	0.699	0.916	0.922
	2	0.590	0.873			0.764	0.910	
	3	0.727	0.855			0.750	0.911	
	4	0.804	0.845			0.830	0.903	
	5	0.835	0.841			0.795	0.906	
	6	0.644	0.864			0.812	0.904	
	7	0.599	0.872			0.666	0.920	
Recovery self‐efficacy	1	0.800	0.924	0.933	0.885	0.584	0.922	0.914
	2	0.840	0.915			0.802	0.893	
	3	0.489	0.955			0.790	0.895	
	4	0.865	0.913			0.763	0.899	
	5	0.903	0.909			0.787	0.895	
	6	0.964	0.899			0.833	0.889	
Total scale	24			0.908	0.916			0.926

*Note:* All ICC are significant at *p* < 0.001 level.

Abbreviation: ICC, intra class coefficient.

## 4 Discussion

This study was undertaken to develop and psychometrically assess the exercise self‐efficacy tool based on the HAPA in heart failure patients undergoing cardiac rehabilitation. Cronbach's alpha exceeding 0.8 for each factor. These outcomes affirm the tool's high reliability.

EFA confirming favorable item commonality. This approach yielded four factors for self‐efficacy, categorized in alignment with the shared content of the questions and the HAPA framework.

Coping self‐efficacy emerged as the initial factor, reflecting individuals’ confidence in their ability to sustain behavior in challenging situations. Patients who initiate rehabilitation encounter competing conditions and must be empowered to surmount exercise‐related barriers and challenges. Elevated self‐efficacy scores enhance the likelihood of success in rehabilitation. Furthermore, this tool holds the potential to provide critical insights for tailoring psychological interventions to this patient group, as low self‐efficacy may lead to relapses or program discontinuation. Individuals with high coping self‐efficacy exhibit greater confidence in employing strategies to overcome obstacles and maintain behavior over extended periods.

These findings are supported by the yang et al. [36] wich emphasizes the significance of addressing perceived barriers and fostering self‐efficacy to improve adherence in this patient population.

Consequently, this construct is sometimes called maintenance self‐efficacy [28, 37], given its significant role in upholding rehabilitation behavior for a defined duration. The Cronbach's alpha coefficient for this construct, signifying its high precision and reliability. While numerous studies emphasize the significance of self‐efficacy in improving adherence to exercise rehabilitation programs [28] [38]. this research specifically addresses the identification and measurement of various dimensions of self‐efficacy in heart failure patients. Rather than focusing extensively on these studies, we concentrate on key findings that demonstrate how the developed tool can significantly improve patients' adherence to exercise programs. This focus allows us to clearly articulate the importance of the self‐efficacy tool within the HAPA model framework.

Maintenance self‐efficacy is pivotal in enhancing adherence to cardiac rehabilitation, particularly in chronic heart failure patients undergoing home‐based programs. Findings from the study [39] highlight the role of psychological constructs like self‐efficacy in promoting sustained exercise adherence and improving long‐term health outcomes.

In conjunction with other factors, such as perceived threat and outcome expectancy, task self‐efficacy is pivotal in influencing the intention to engage in sports‐related behaviors during the motivational phase of the HAPA. The study observed that task self‐efficacy could be delineated into two distinct factors. Specifically, items related to an individual's confidence in exercising for shorter durations (i.e., 15 and 20 min) and on fewer days per week (2, 3, and 4 times) were grouped into one factor, termed “task self‐efficacy 1”. Meanwhile, “task self‐efficacy 2” encompassed items assessing individuals' confidence in engaging in longer‐duration exercise sessions (30, 40, 50, and 60 min per day) on a more frequent basis (5 and 6 times a week). Notably, previous studies had typically considered task self‐efficacy as a single factor [28, 38].

The fact that the tool distinguishes between two separate factors for task self‐efficacy suggests the importance of discerning between self‐efficacy for commencing exercise with lower intensity and fewer sessions versus self‐efficacy for commencing exercise with higher intensity and more frequent sessions. Patients tend to experience varying levels of self‐efficacy when initiating rehabilitation, depending on the intensity of their exercise regimen [40]. This self‐efficacy component is the foremost predictor of an individual's intention to commence rehabilitation [41]. The degree to which individuals decide to initiate exercise is closely associated with goal‐setting strategies in their planning [42]. Establishing exercise goals based on time frames enhances self‐efficacy and facilitates program initiation by enhancing their mental preparedness for action. The more congruent this planning is with the patient's sense of empowerment, the more likely it will be implemented or adhered to by the patient. Individuals with high task self‐efficacy are more inclined to engage in new behaviors [43]. These result affirm the tool's reliability, as it demonstrated strong consistency in measuring task self‐efficacy before the commencement of rehabilitation.

Recovery self‐efficacy, assesses individuals' confidence levels in their ability to resume rehabilitation following a relapse. This factor gauges the extent of people's confidence in their capability to return to the rehabilitation program should they experience an interruption in their treatment. Individuals who perceive relapse as a failure are less likely to persist with their treatment, as opposed to those who maintain faith in their capacity to recover. Numerous studies have consistently highlighted the substantial impact of improved self‐efficacy on the durability and prevention of relapses in cardiac rehabilitation among patients recovering from myocardial infarctions [13, 19, 44]. The internal consistency of this construct, as measured by Cronbach's alpha, indicating excellent reliability, a result consistent with Rouhani's study.

Patients embarking on cardiac rehabilitation must commit to extended periods of exercise and adapt their lifestyles to harness the benefits of physical activity, thereby enhancing cardiac performance. The risk of relapse in this context is notably high. Consequently, the role of recovery self‐efficacy assumes excellent significance, as it can serve as a means to steer patients back onto the treatment path. Accordingly, this construct was designed within the HAPA behavior change stages framework, although some prior studies may not have incorporated it [45, 46].

## 5 Limitation

This study has several limitations that should be considered. The first limitation is sample size, which may affect the generalizability of the results. Since our sample consists of heart failure patients, these findings may not be applicable to other populations or medical conditions.

The second limitation concerns cultural issues associated with the Iranian population. When assessing self‐efficacy and exercise behaviors, it is essential to examine cultural and social factors carefully, as these can significantly influence patients' perceptions of self‐efficacy and their participation in rehabilitation programs.

Finally, future research should explore these concepts in more diverse populations to gain a better understanding of the cultural and social influences on self‐efficacy and exercise behaviors.

## 6 Conclusion

The Cardiac Rehabilitation Self‐Efficacy Questionnaire was specifically developed and validated to assess exercise self‐efficacy constructs among heart failure patients undergoing rehabilitation, considering the psychological profiles of these individuals within the framework of the HAPA. Initially conceptualized with four distinct factors, this tool has exhibited high validity, reliability, and substantial item commonalities. Consequently, it can be effectively employed in research endeavors to elucidate patients’ self‐efficacy levels during the rehabilitation stages of the HAPA model. The utility of this tool extends to the creation of tailored interventions designed to bolster patient self‐efficacy and their subsequent integration into HAPA‐related interventions.

## Author Contributions

**Zahra Fallah:** conceptualization, writing–original draft, visualization, writing–review and editing. **Awat Feizi:** methodology, software, data curation, writing–review and editing. **Masoumeh Sadeghi:** conceptualization; writing–original draft; writing–review and editing; visualization. **Mohammad Mahdi Hadavi:** writing–review and editing; writing–original draft; validation; investigation. **Hossein Shahnazi:** conceptualization; funding acquisition; writing–original draft; writing–review and editing; validation; project administration; supervision; resources.

## Conflicts of Interest

Authors declare no conflicts of interest.

## Transparency Statement

The lead author Hossein Shahnazi affirms that this manuscript is an honest, accurate, and transparent account of the study being reported; that no important aspects of the study have been omitted; and that any discrepancies from the study as planned (and, if relevant, registered) have been explained.

## Data Availability

All authors have read and approved the final version of the manuscript. Corresponding author had full access to all of the data in this study and takes complete responsibility for the integrity of the data and the accuracy of the data analysis.
